# Differential methylation region detection via an array-adaptive normalized kernel-weighted model

**DOI:** 10.1371/journal.pone.0306036

**Published:** 2024-06-28

**Authors:** Daniel Alhassan, Gayla R. Olbricht, Akim Adekpedjou

**Affiliations:** Department of Mathematics and Statistics, Missouri University of Science and Technology, Rolla, MO, United States of America; UC Los Angeles: University of California Los Angeles, UNITED STATES

## Abstract

A differentially methylated region (DMR) is a genomic region that has significantly different methylation patterns between biological conditions. Identifying DMRs between different biological conditions is critical for developing disease biomarkers. Although methods for detecting DMRs in microarray data have been introduced, developing methods with high precision, recall, and accuracy in determining the true length of DMRs remains a challenge. In this study, we propose a normalized kernel-weighted model to account for similar methylation profiles using the relative probe distance from “nearby” CpG sites. We also extend this model by proposing an array-adaptive version in attempt to account for the differences in probe spacing between Illumina’s Infinium 450K and EPIC bead array respectively. We also study the asymptotic results of our proposed statistic. We compare our approach with a popular DMR detection method via simulation studies under large and small treatment effect settings. We also discuss the susceptibility of our method in detecting the true length of the DMRs under these two settings. Lastly, we demonstrate the biological usefulness of our method when combined with pathway analysis methods on oral cancer data. We have created an R package called *idDMR*, downloadable from GitHub repository with link: https://github.com/DanielAlhassan/idDMR, that allows for the convenient implementation of our array-adaptive DMR method.

## Introduction

DNA methylation is an important epigenetic mechanism used by cells to control gene expression [1]. It plays an important role in many biological processes such as somatic cell [2] and embryonic development [3]. Aberrant DNA methylation patterns have been linked to complex diseases like cancer and diabetes [4]. DNA methylation refers to the addition of a methyl group to a DNA base. In mammals, it is known to occur at cytosine sites when followed by a guanine nucleotide (called a CpG site) [5]. Whole-genome bisulfite sequencing (WGBS) is the gold standard for measuring methylation status in any organism. It can capture more than 28 million CpGs [6], providing genome-wide coverage, whereas microarrays focus on a subset of the genome, targeting specific genomic regions. Despite the massive reduction in cost of WGBS in recent years, it is still expensive when employed in large-scale epidemiological studies. Microarrays have become more popular for most epigenome-wide association studies (EWAS). They provide an economically feasible [7] means to explore the associations between DNA methylation and complex diseases [8]. These studies aim to better understand the connection between DNA methylation and human health through identifying markers associated with diseases.

Illumina Infinium HumanMethylation BeadChip technology is the most widely used array-based technology for EWAS studies. The Illumina platform estimates the methylation status using two probes (methylated and unmethylated) at each CpG site to measure the methylation intensities [9]. There are two ways of quantifying methylation output from the Illumina BeadChip assay: (1) the *β*-value and (2) the M-value. The *β*-value measures the percentage of methylation and hence ranges from 0 (unmethylated) to 1 (fully methylated). The M-value is calculated from the *β*-value using the following relationship:

$$\begin{array}{c} M={\text{log}}_{2}(\frac{\beta }{1-\beta }). \end{array}$$ (1)

Though the *β*-value is biologically preferred when it comes to interpretation, the M-value is statistically more appropriate. Most classical statistical methods, like the general linear model, used in analyzing high-throughput experiments assume equal variances of populations and normality of errors. The M-values approximately have equal variance and have the same support as the Gaussian distribution. Thus, a statistic based on M-values is more appropriate when using such methods [10].

Over the years, Illumina has been improving their DNA methylation assay by increasing the number of CpG sites that can be interrogated, starting with the Infinium HumanMethylation 27K (∼27,000 CpG sites), Infinium HumanMethylation 450K (∼480,000 CpG sites) to the most recent Infinium HumanMethylationEPIC (∼850,000 CpG sites). While the Infinium HumanMethylationEPIC array (herein termed ‘EPIC’) represents the latest advancement in methylation assays, the Infinium HumanMethylation 450K array (herein termed ‘450K’) continues to hold significant relevance. Historically, the 450K array was one of the first to provide comprehensive coverage of methylation sites across the genome, making it foundational in epigenetic research. Its widespread adoption has led to the accumulation of a large volume of data over years, which now serves as a critical benchmark for longitudinal studies [11, 12].

As the technology evolves, future arrays may have different probe gap distributions and a method the readily adapts to the type of array is needed. In this article, we will focus on 450K array data but also provide a method that adapts to the EPIC and possibly future Infinium assays. An important characteristic of the two arrays (450K and EPIC) is that the methylation intensities are measured using either the Infinium I assay or Infinium II assay which have different chemistries. For a detailed description of these two assays, see [13] and references therein. Owing to the different chemistries but complimentary strengths of the two designs, data preprocessing and normalization are critical [14]. Several normalization methods [15–17] exist in the literature and though no single one always outperforms the other, some methods are built ideally for some specific cases. For instance, the functional normalization method [15] is best suited for cases where global differences are expected, such as in treatment-control studies. We employed this normalization technique in our simulation and data application example.

Differences in DNA methylation between samples (e.g. cancer and normal) can be measured at single CpG sites, referred to as differentially methylated loci (position) (DML/DMP), and over contiguous sites, referred to as differentially methylation regions (DMRs). Despite the many situations where researchers are interested in site-level testing [18], a more useful form is one that involves testing over a region due to the increase in statistical power and biological interpretation [19, 20]. Methylation status between nearby CpG sites are highly correlated (co-methylated) [21, 22] and this information is employed when collapsing contiguous sites to form DMRs. Regions could be either predefined or user-defined. Differential DNA methylation in predefined regions based on genomic annotations such as CpG Islands (CGI), TSS200 (regions from transcription start site to 200 bases upstream), TSS1500 (200–1500 bases upstream of the TSS), Open sea (regions of the genome with sparse CpG density, distal from CpG islands), and CGI shores (regions immediately adjacent to CpG Islands, typically showing a gradient in CpG density) have special biological interpretations [23–26]. However, they possess two problems: (1) they comprise only a subset of the 450K/EPIC probes for DMR detection which provide less room for knowledge discovery and (2) they require defining genomic regions prior to evaluation, forcing DMRs to have artificial start and end points. A user-defined region, however, allows for flexibility and knowledge discovery as regions can be defined based on some criteria such as median distance between probes. The method we propose falls within this group.

Many user-defined DMR detection methods such as *Probe Lasso*, *Bump Hunter*, *DMRcate* among others, have been proposed [27–31]. However, no one approach always outperforms the other. The *Probe Lasso* method [28] capitalizes on the uneven spacing of probes based on genomic annotation on the array. It calls DMRs based on the probe density so that subsequent analysis do not entirely focus on the dense regions alone. Though the DMR calling framework is purported to be dynamic or flexible, it still forces DMRs to have artificial start and end points within a gene feature. It is a user-defined region method between gene features but a predefined region method within gene features, hence it may fail to detect other novel DMRs when they do exist (lack statistical power). *Bump Hunter* [29], employs surrogate variable analysis to handle batch effects, a unique feature in their method, as samples are usually not collected at the same time point. However, in an extensive simulation study under 60 different parameter settings [32] comparing the popular methods (*Probe Lasso* and *DMRcate* included), it was revealed that *Bump Hunter* was slow, lacked power (under large and small effect size) and ranked last in terms of precision. *DMRcate* [31] is a novel method that only uses the spatial distribution of probes to call DMRs and given a window, borrows information from nearby CpG sites using a Gaussian kernel. *DMRcate* has gained much popularity in the literature due to its particularly superior predictive performance compared to *Probe Lasso* and *Bump Hunter* [32]. Despite this success, there is still a higher tendency to incur bias due to irregularly spaced CpG sites and further lack the ability to detect all true DMRs that may exist. Given a specific window, highly dense regions are more likely to be detected as all nearby CpG sites will each receive weights that are very close to one. However, less dense regions, may receive no weights at all. This bias towards denser regions leads to the high detection of DMRs in those regions but also a low sensitivity in detecting true DMRs that may exist in less dense regions.

Furthermore, the high weights and subsequently smoother estimates obtained in the high dense regions is not entirely realistic as it does not account for the contribution of each nearby CpG site in obtaining the smoothed estimate. That is, if three adjacent sites *A*, *B*, and *C* are considered neighbors because they are within some genomic distance of each other, then we must consider the relative contribution from these neighbors rather than the raw contribution when attempting to smooth site-level statistics at *A*. Accounting for the relative contribution could reduce the bias in detecting DMRs to a level sufficient to detect true DMRs in the high dense regions while also improving statistical power to detect DMRs in less dense regions.

Another limitation of the previous methods is that they do not sufficiently address varying patterns of co-methylation (similar methylation profiles) in different genomic regions. [21] first identified a significant correlation between methylation levels and CpG distance within a span of 1000 base pairs (bp), a foundational finding cited by many DMR detection methods including the aforementioned. However, recent studies show that co-methylation varies across the genome. For instance, [33] indicates that co-methylation can occur within distances as short as a few hundred base pairs in normal tissues. Further analysis by [34] in a breast cancer study revealed significant variations in co-methylation region lengths between unmethylated and methylated states. In a comprehensive follow-up, [35] also examined co-methylation patterns in breast cancer data, uncovering distinct patterns on chromosome X compared to other chromosomes.

In addition to the recent findings on co-methylation, the low statistical power due to the bias and lack of flexibility in the aforementioned methods suggest the need for a much more flexible and less-biased DMR detection method. Furthermore, the continued research in this field evidenced by other recently developed methods by [36, 37] have further underscored the critical relevance of DMR studies using microarray data. To this end, the goals of this manuscript are three-fold: (1) we propose a general locally-weighted statistic via which co-methylation information can be incorporated to site-level statistics for DMR detection, (2) we show some large sample properties of statistics of this form under some regularity conditions and (3) we develop a new method for DMR detection (a specific case of the general locally-weighted statistic), which reduces the bias due to irregular spaced CpG sites and increases the sensitivity in detecting true DMRs. Furthermore, we formulate an array adaptive version of the method (*aaDMR*) to better capture the somewhat complex co-methylation among CpG sites, and that adapts to the spacing on each chromosome for 450K, EPIC and possibly future arrays.

## Methods

### Site-level differential methylation testing with limma

Limma [38] stands for “linear models for microarray data” and it is the most widely used method for microarray analysis. It involves fitting linear models and is commonly used in the analysis of DNA methylation data. We use limma for our work to obtain differential methylation signals at individual CpG sites. To this end, consider testing *H*_0_: *μ*_*T*_ − *μ*_*N*_ = 0 against *H*_1_: *μ*_*T*_ − *μ*_*N*_ ≠ 0 where *μ*_*T*_, *μ*_*N*_ are average methylation M-values from tumor and normal samples respectively at a CpG site obtained from the respective true percent of methylation *β*_*T*_ and *β*_*N*_ based on Eq (1). We fit a linear model with the M-values as the response, the condition (tumor or normal) as the predictor, along with any covariates of interest. The specific contrast to test the above hypotheses is conducted and the empirical Bayes techniques is then implemented to obtain robust *t* estimates called moderated *t*-statistics. The DMR detection methods we discuss below rely on the robust site-level tests from limma.

### A general locally-weighted statistic

DNA methylation levels are correlated, at least for nearby CpG sites [21, 22]. Hence in determining the DMRs, we propose smoothing limma’s moderated *t*-statistic as a suitable way to capture the correlation information among nearby CpG sites. To this end, we propose the general locally-weighted statistic Eq (2), motivated by *DMRcate*’s kernel-weighted statistic. For a chromosome, define the locally weighted statistic *S*(*x*_*i*_) as:

$$S(x_{i})=Y_{i}+\sum_{j\neq i}^{n}w_{j}\left(x_{i}\right)Y_{j}$$ (2)

where *x*_*i*_ denotes the position of CpG site *i* (site of interest where smoothing is happening) and *j* (neighboring sites) respectively, *n* is the number of sites within some specified genomic distance, *Y*_*i*_(or *Y*_*j*_) denotes some function of a statistic from a site-level testing (such as the moderated *t*-statistic from limma [38]), and *w*_*j*_(*x*_*i*_), some appropriate weighting function or mechanism, used to account for the interdependencies between nearby CpG sites. For the purposes of our work, we define *Y* = *T*^2^ where *T* is a random variable representing limma’s moderated *t*-statistic [38]. We state in passing, that when *w*_*j*_(*x*_*i*_) = *K*_*ij*_, defined as Gaussian kernel weights as in [31], we obtain *DMRcate’s* kernel-weighted statistic *S*_*KY*(*i*)_. The EPIC array has ∼400,000 CpG sites more than 450K array and so within some specified genomic distance, EPIC is likely to contain more sites than the 450K. With this in mind, we investigate the asymptotic behavior of *S*(*x*_*i*_) when the number of nearby CpG sites increases within some specified genomic distance of *x*_*i*_ (or as the array technology improves). Investigating the asymptotic behavior of our statistic is crucial as it guarantees the accuracy and reliability of our results, providing a solid foundation for our conclusions in the complex study of DNA methylation. For readers interested in the detailed mathematical underpinnings of these theorems, extensive proofs and derivations are provided in the supplementary text (see S1 File).

### Normalized kernel-weighted statistic

We propose a specific weighting function *w*_*j*_(*x*_*i*_) (simply *w*_*j*_) called the normalized kernel-weight function (3) as a realistic way of incorporating the interdependencies among nearby CpG sites. Kernel smoothers allow for a flexible degree of smoothing via its smoothing parameter, known as the *bandwidth*, which is an effective way of incorporating the shared methylation profiles at nearby CpG sites. Our rationale for the normalized kernel is two fold. First, if *A* < *B* < *C*, where *A*, *B*, *C* are CpG sites, then within some “reasonable genomic distance” (determined by the bandwidth), if *A* is a neighbor to *B*, and *B* a neighbor to *C*, then *A* and *C* are neighbors as well. So in smoothing or weighting the statistic at *A* for instance, one needs to consider the relative contribution from the nearby CpG sites since there is shared co-methylation among the neighbors. Secondly, the NK reduces the bias towards dense regions in the DMR detection process through a “fair” distribution of the weights thereby increasing the sensitivity in detecting DMRs in less dense regions when they do exist. With the caveat that the total of all contributed weights must equal one, our method maintains the property that sites closer to the reference site contribute more weight than those further away. One major difference between our proposed method and DMRcate lies in the rationale behind the statistics proposed. We hypothesize that within some genomic distance all neighboring sites contain the totality of information to smooth a site-level statistic. However, *DMRcate* advocates for using raw contributions from neighboring sites in smoothing a site-level statistic. As previously mentioned, DMRcate procedure is biased to CpG dense regions due to their statistic [32]. The major advantage in our approach is the increase sensitivity or power in picking up DMRs that do exist in less dense regions while maintaining our precision in picking up DMRs in CpG-dense regions. For convenience, we employ the Gaussian kernel, $K\left(z\right)=\text{exp}(-\frac{z^{2}}{2})$, and define the weighting function as:

$$\begin{array}{c} w_{j}\left(x_{i}\right)=\frac{K(\frac{x_{j}-x_{i}}{h})}{\sum_{j\neq i}^{n}K(\frac{x_{j}-x_{i}}{h})}, \end{array}$$ (3)

where *K*(⋅) is the kernel, *h* is the bandwidth and *x*_*i*_, *x*_*j*_ denote the position of CpG sites *i* and *j* respectively. More specifically, we address the complex co-methylation patterns in the manner stated below.

**Fig 1:**
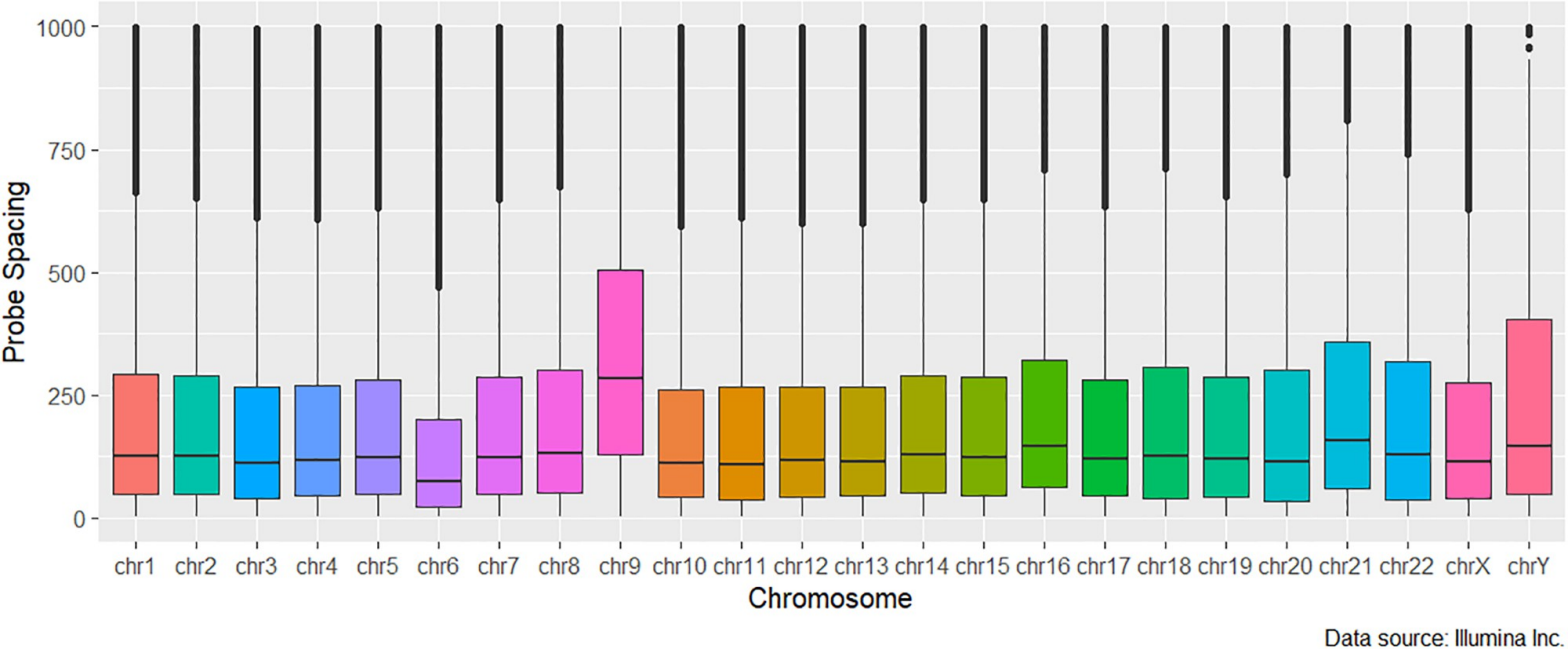
Probe spacing distribution on the 450K array truncated at 1000bp to ease visualization.

**Fig 2:**
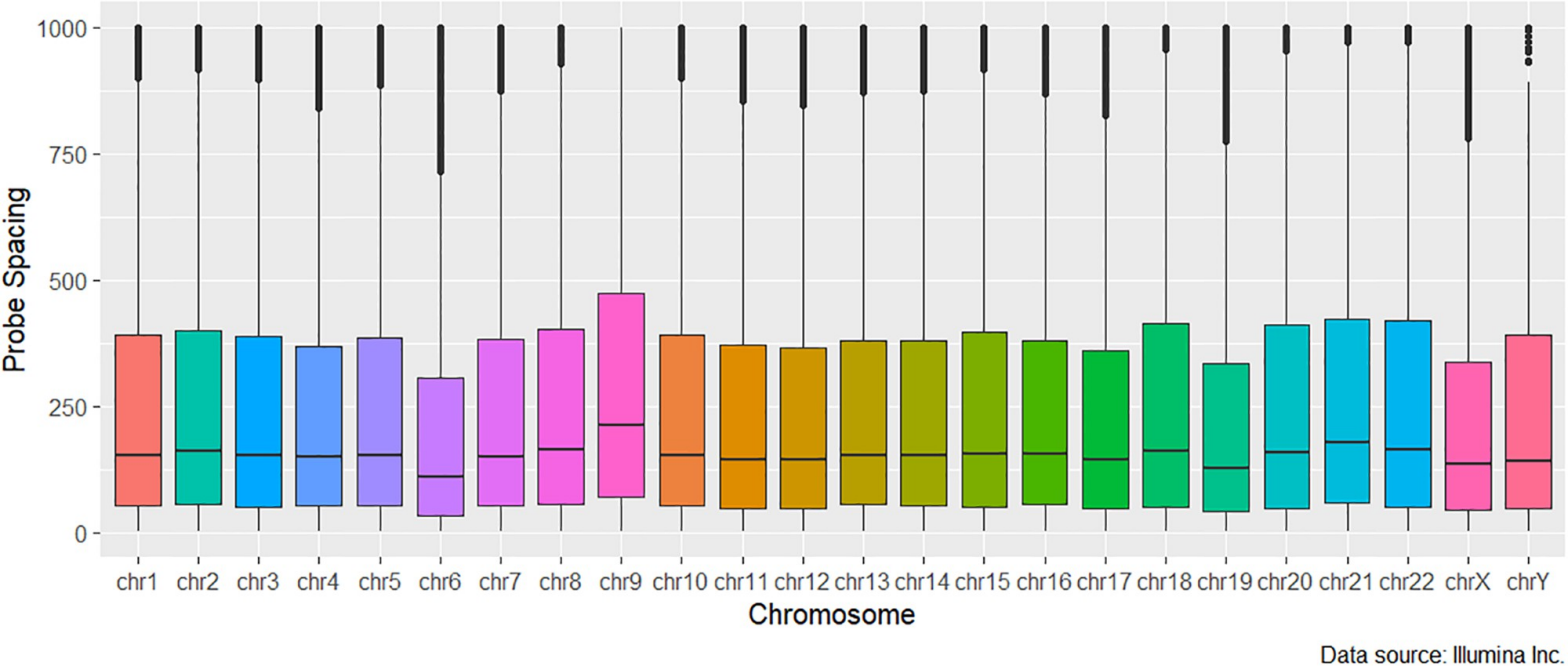
Probe spacing distribution on the EPIC array truncated at 1000bp to ease visualization.

(i) First, we employ the normalized kernel-weight Eq (3) to address the interdependencies in the methylation levels at nearby sites. In order to readily capture the benefit gained by using our weighting measure, we keep a fixed bandwidth/ kernel size, *h* of 500bp, the optimal bandwidth used in [31]. We call this approach the fixed-spacing array DMR (*faDMR*) detection method.
(ii) Our first approach essentially assumes equal spacing between the probes on each chromosome which is far from truth. See Figs 1 and 2 for the distribution of probe gaps on the 450K and EPIC respectively. In addition to the uneven spacing at the chromosomal level, co-methylation patterns are different for different chromosomes [35], suggesting that using a different kernel size, *h*, for each chromosome may prove useful, since *h* acts as a measure of spread. To that end, we propose an array-adaptive DMR (*aaDMR*) detection method, one that chooses *h* to equal the median probe spacing on each chromosome.

### Modeling *S*(*x*_*i*_) via Satterthwaite’s approximation

We describe the process of modeling our normalized kernel-weighted statistic and mention that the process is similar to that used by *DMRcate* [31]. Let *x*_1_ < *x*_2_, ⋯, <*x*_*n*_, be CpG site positions for an individual chromosome. Under the appropriate null hypothesis, *T* ∼ *t*_*ν*_ where *ν* is the degrees of freedom (after empirical Bayes’ adjustment) so that *Y* ∼ *F*_(1,*ν*)_. It is obvious that Eq (2) is a weighted linear combination of F-distributed random variables which is mathematically complex to model [31]. Owing to the empirical Bayes method used by limma [38], *ν* is relatively large even for small sample size situations. We capitalize on this so that as *ν* → ∞, $v\overset{d}{\to }x_{1}^{2}$.

We can now view Eq (2) as a linear combination of scaled $\chi_{1}^{2}$ random variables. Assuming that *S*(*x*_*i*_) is approximately distributed as a scaled chi-squared random variable of the form $p_{x_{i}}\chi_{q_{x_{i}}}^{2}$, we utilize the rule suggested by Satterthwaite [39] by matching the first two central moments in Eq (4) to obtain $p_{x_{i}}$ and $q_{x_{i}}$ in Eq (5). The first two central moments of *S*(*x*_*i*_) are $E\left(S\left(x_{i}\right)\right)=E(Y_{i}+\sum_{j=1}^{n}w_{j}Y_{j})=1+\sum_{j=1}^{n}w_{j}$ and $Var\left(S\left(x_{i}\right)\right)=Var(Y_{i}+\sum_{j=1}^{n}w_{j}Y_{j})=2(1+\sum_{j=1}^{n}w_{j}^{2})$ respectively. We adopt the notation $p_{x_{i}}$ and $q_{x_{i}}$ to make clear that the constants are obtained on a CpG-site-level and hence may differ from one site to another. The mean and variance of $p_{x_{i}}\chi_{q_{x_{i}}}^{2}$ are given by $p_{x_{i}}q_{x_{i}}$ and $2p_{x_{i}}^{2}q_{x_{i}}$ respectively. By matching the first two central moments to the mean and variance stated, we have:

$$\{\begin{array}{ll} p_{x_{i}}q_{x_{i}} & =1+\sum_{j=1}^{n}w_{j}\\ 2p_{x_{i}}^{2}q_{x_{i}} & =2(1+\sum_{j=1}^{n}w_{j}^{2}). \end{array}$$ (4)

Solving (4) leads to

$$\{\begin{array}{l} p_{x_{i}}=\frac{1+\sum_{j=1}^{n}w_{j}^{2}}{1+\sum_{j=1}^{n}w_{j}}\\ q_{x_{i}}\frac{{\left(1+\sum_{j=1}^{n}w_{j}\right)}^{2}}{1+\sum_{j=1}^{n}w_{j}^{2}} \end{array}$$ (5)

Now, since $S(x_{i})∼p_{x_{i}}\chi_{q_{x_{i}}}^{2}$ then, $\frac{S(x_{i})}{p_{x_{i}}}∼\chi_{q_{x_{i}}}^{2}$. We compare the observed values of $\frac{S(x_{i})}{p_{x_{i}}}$ to a *χ*^2^ distribution with $q_{x_{i}}$ degrees of freedom to obtain p-values for our local estimator. Next, we apply the Benjamini-Hochberg (BH) [40] correction to control the false discovery rate (FDR) across all site-level tests. CpG sites with a BH-corrected p-value less than the significance level of *α* = 0.05 are retained. As a last step, we group significant CpG sites (retained from the previous procedure) that are within *g* genomic distance from each other to form DMRs. In collapsing these contiguous sites into regions, we defaulted to *g* = 1000 bp as do DMRcate [31] and Bumphunter [29]. To quantify statistical uncertainty to the identified DMRs, we take the CpG site with the minimum p-value as a representative p-value for that region.

### Step-by-step summary of the faDMR and aaDMR detection methods

(a) Obtain site-level moderated observed *t*-statistics, *t*_*j*_, using limma [38].
(b) Calculate observed $y_{j}=t_{j}^{2}$ for CpG site *j*.
(c) Use the normalized kernel weight Eq (3) to weight observed *y*_*j*_ to obtain smoothed locally weighted statistic *S*(*x*_*i*_) Eq (2). For the faDMR method, *h* is set to 500bp. For the aaDMR method, *h* is taken to equal the median probe spacing on each chromosome.
(d) Use Satterthwaite’s approximation to model *S*(*x*_*i*_) to obtain unadjusted p-values.
(e) Apply BH correction to obtain adjusted p-values.
(f) Filter out any CpG site with an adjusted p-value greater than *α* = 0.05.
(g) Specify *g*, the agglomerate parameter, and collapse significant sites from (f) that are within *g* base pairs of each other to form DMRs.
(h) Take the minimum p-value as a representative p-value across CpG sites in the region. This p-value is used to order the regions in terms of the strength of significance.

## Results

### Simulation study

**Fig 3:**
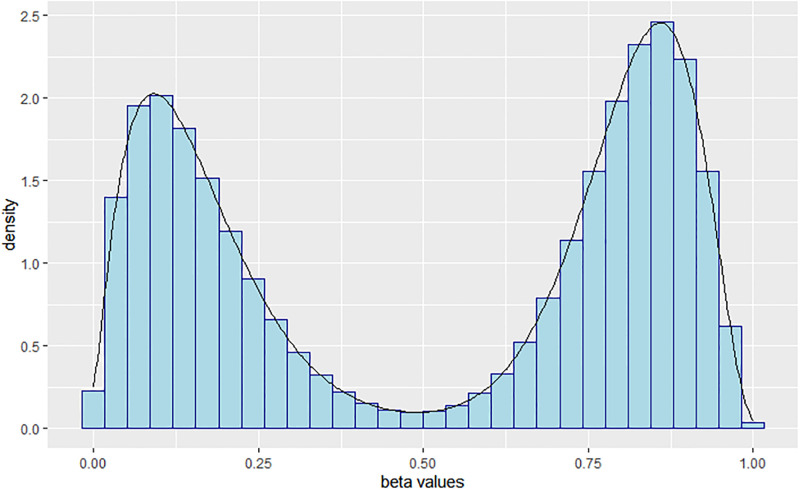
Distribution of *β*–values from one of 1000 datasets showing the parameter space is well explored.

To validate our method and investigate its performance, we perform a simulation study and compare our method with *DMRcate*. We simulated 1000 repetitions of a 450K data set, each with 10 control and 10 treatment samples yielding a 450K × 20 matrix. In each set of simulated data, we randomly assigned 2,136 promoter regions comprising TSS200 and TSS1500 as true DMRs out of 21,363 regions. Half of these were hypermethylated (i.e., methylation higher in treatment than control) and the other half hypomethylated (i.e., methylation lower in treatment than control). For DMRs, *β*–values were simulated from a beta distribution with mode equal to some specified beta level. For non-DMRs, probes were classified as completely methylated or unmethylated based on array data from The Cancer Genomic Atlas (TCGA) on cholangiocarcinoma (CHOL) [41]. For non-DMRs, *β*–values were simulated from a beta distribution with parameters manually chosen to match the average modes of two methylation statuses of CHOL data. A detailed description of the simulation study can be found in the supplementary text (see S1 File). We investigated two different treatment effects (large and small). For the large effect, we set the true methylation difference (Δ*β* = 0.2) to be exactly 0.2 (as in *DMRcate* [31]). However, [32] reported that the common DMR testing methods such as *DMRcate*, lacked power to detect small effect sizes. Consequently, we compared our methods to *DMRcate* with a true methylation difference of 0.09 (small effect) while maintaining other aspects of the simulation unchanged. All analyses were based on the M-values (see (1)) Our simulation study was set up in a similar way to *DMRcate*. In addition, we obtained a histogram for one of the 1000 datasets to investigate how well the parameter space is explored (see Fig 3).

### Evaluation criteria

We compare our methods (*faDMR* and *aaDMR*) with *DMRcate* under its best (default) performance setting (see [31]) to assess their performance using three criteria (precision, recall and F1-score). As previously stated, we compare *faDMR* to *DMRcate* to readily capture the benefit gained from using the normalized kernel. Next we compare *faDMR* and *aaDMR* to capture the advantage of using a bandwidth that adapts to the array (and chromosome). We consider two forms of overlap in each criteria that follows: (1) equal overlap (EO)—where start and end positions of significant DMRs match that of true DMRs; (2) any overlap (AO)—where significant DMRs intersect true DMRs. When a significant DMR from any method does not overlap a true DMR, we call that a false positive (FP). Similarly, we define a true positive as an overlap between a significant DMR and a true DMR. When a true DMR is not significant, we call it a false negative (FN). The three criteria we use to evaluate performance are:

(i) Recall (power): We estimate power by dividing the number of true positives (TP) by (TP + FN), ie. 2136 true DMRs. That is, recall = $\frac{TP}{TP+FN}$.
(ii) Precision: We estimate precision by dividing the number of true positive DMRs by (TP + FP), i.e. the total number of significant DMRs found by our methods. That is, precision = $\frac{TP}{TP+FP}$.
(iii) F1 score: This metric combines precision and recall into a single metric ranging from 0 to 1, where 1 represents perfect precision and recall. Some methods have a tendency to prioritize reducing false positives (increasing precision) at the expense of recall (more false negatives). We use this metric, which assigns equal weight to precision and recall, because relying solely on either precision or recall does not provide a comprehensive assessment of the performance of the method. We compute F1 score as, $F_{1}=2\times \frac{\text{precision}\times \text{recall}}{\text{precision}+\text{recall}}$ [32, 42].

### Simulation results

Based on the EO criteria (see Table 1), the type I error rates (i.e., percent of FPs) for our methods are around the expected 5% (5.16% for *faDMR* and 5.35% for *aaDMR*) with DMRcate a little above (6.07%). The story is reversed using the AO criteria (see Table 2) with all methods well controlling the type I error rate at less than 5% (0 FP for *DMRcate*, 2 FPs for *faDMR* and 1 FP for *aaDMR*). Both *faDMR* and *aaDMR* detected substantially more DMRs compared to DMRcate from of a total of 2136 (see Tables 1 and 2).

**Table 1:** Large treatment effect (Δ*β* = 0.2): A confusion matrix comparing the results from three methods (DMRcate, faDMR and aaDMR) with true DMRs based on EO criteria averaged across 1000 datasets. Sig. means statistically significant DMRs; Not Sig. indicates the number of regions that are not statistically significant.

DMRcate	True DMR	No DMR	Total
Sig	**284**	1168	**1452**
Not Sig.	1852	18059	19911
Total	2136	19227	21363
faDMR	True DMR	No DMR	Total
Sig.	**753**	992	**1745**
Not Sig.	1383	18235	19618
Total	2136	19227	21363
aaDMR	True DMR	No DMR	Total
Sig.	**772**	1030	**1802**
Not Sig.	1364	18197	19561
Total	2136	19227	21363

**Table 2:** Large treatment effect (Δ*β* = 0.2): A confusion matrix comparing the results from three methods (DMRcate, faDMR and aaDMR) with true DMRs based on AO criteria averaged across 1000 datasets. Sig. means statistically significant DMRs; Not Sig. indicates the number of regions that are not statistically significant.

DMRcate	True DMR	No DMR	Total
Sig	**1452**	0	**1452**
Not Sig.	684	19227	19911
Total	2136	19227	21363
faDMR	True DMR	No DMR	Total
Sig.	**1743**	2	**1745**
Not Sig.	393	19225	19618
Total	2136	19227	21363
aaDMR	True DMR	No DMR	Total
Sig.	**1801**	1	**1802**
Not Sig.	335	19226	19561
Total	2136	19227	21363

**Fig 4:**
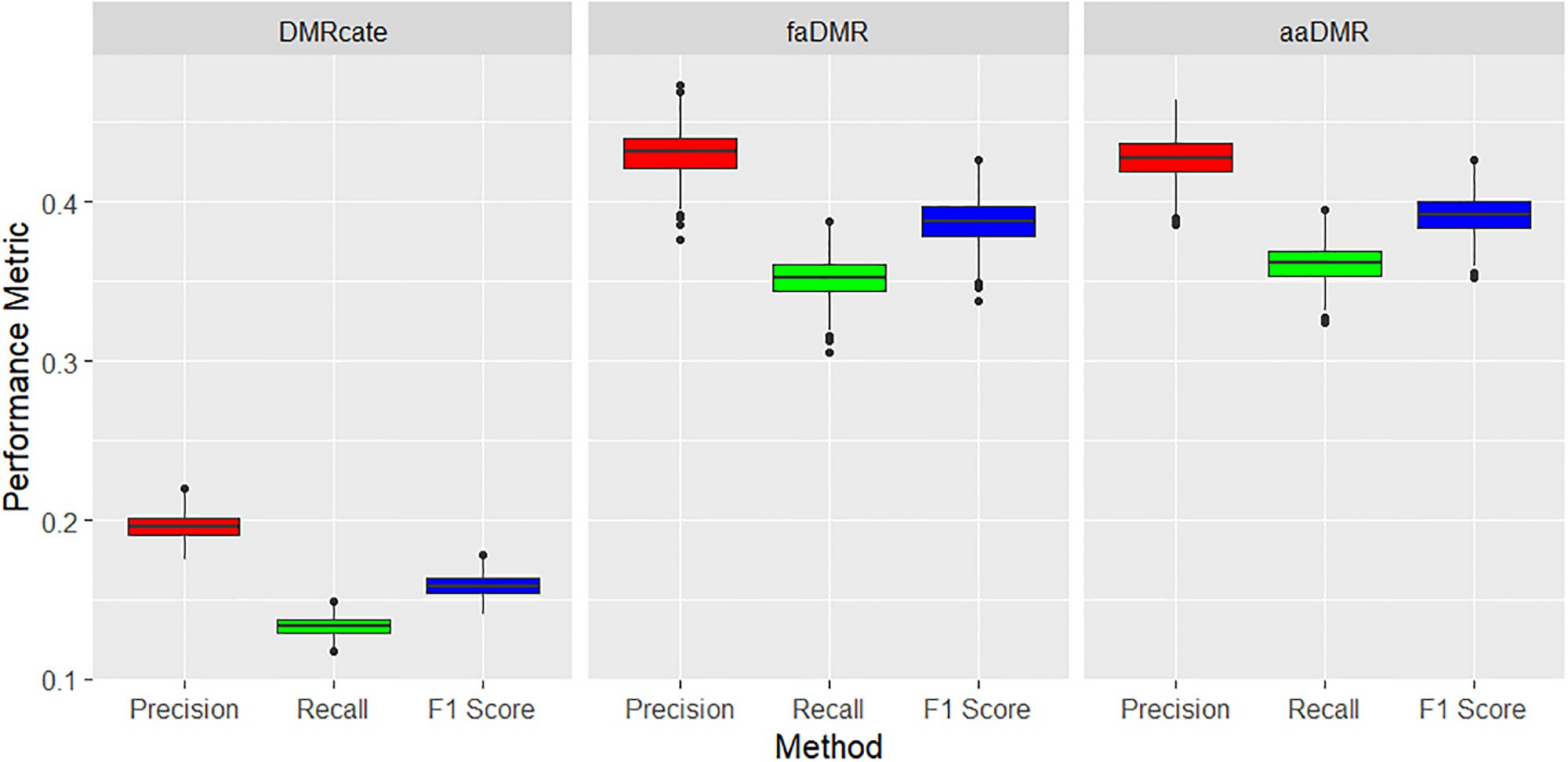
Large treatment effect (Δ*β* = 0.2): Precision, recall and F1 score metrics based on EO criteria. Boxplots of results across the 1000 simulated data sets. All pairwise comparisons of the methods are statistically significant with p < 0.001 using Tukey’s multiple comparison test from a one-way ANOVA.

**Fig 5:**
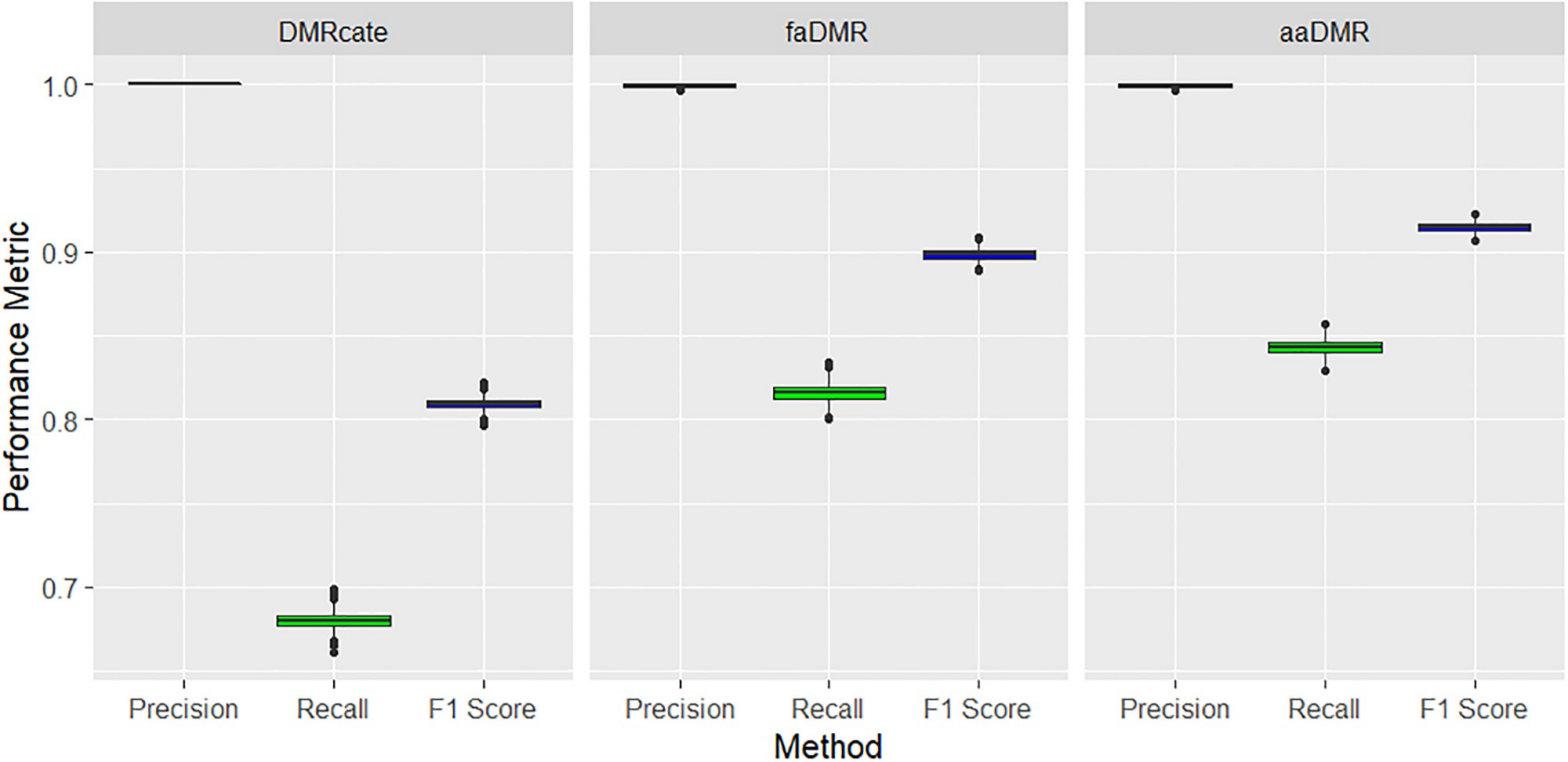
Large treatment effect (Δ*β* = 0.2): Precision, recall and F1 score metrics based on AO criteria. Boxplots of results across the 1000 simulated data sets. All pairwise comparisons of the methods are statistically significant with p < 0.001 using Tukey’s multiple comparison test from a one-way ANOVA.

The precision, power and F1-score metrics all indicate *faDMR* and *aaDMR* methods are outperforming *DMRcate*. Based on the EO criteria, DMRcate performed poorly on precision, power and F1 score with median of less than 0.2 on all three criteria across the 1000 simulated data sets. Though power and precision are less than 50% for all methods, it’s readily improved with median values in *faDMR* and *aaDMR* (averaged across 1000 datasets) more than twice that of *DMRcate* (see Fig 4). At the time of writing, we have not come across an article in this domain that utilizes the EO criteria. Most authors tend to favor the less strict AO criteria [32]. Based on the AO criteria, all three methods perform well in terms of precision with DMRcate sacrificing power (less than 0.7) for nearly perfect precision. *faDMR* and *aaDMR* both improve power (between 0.8 and 0.9) without hurting precision (see Fig 5). In allowing for the array-adaptive case, we were able to slightly improve power by 3% (comparing *faDMR* to *aaDMR* under AO criteria). Comparing *faDMR* and *aaDMR* using the EO criteria, we notice a slight increase in power (∼ 1%).

**Fig 6:**
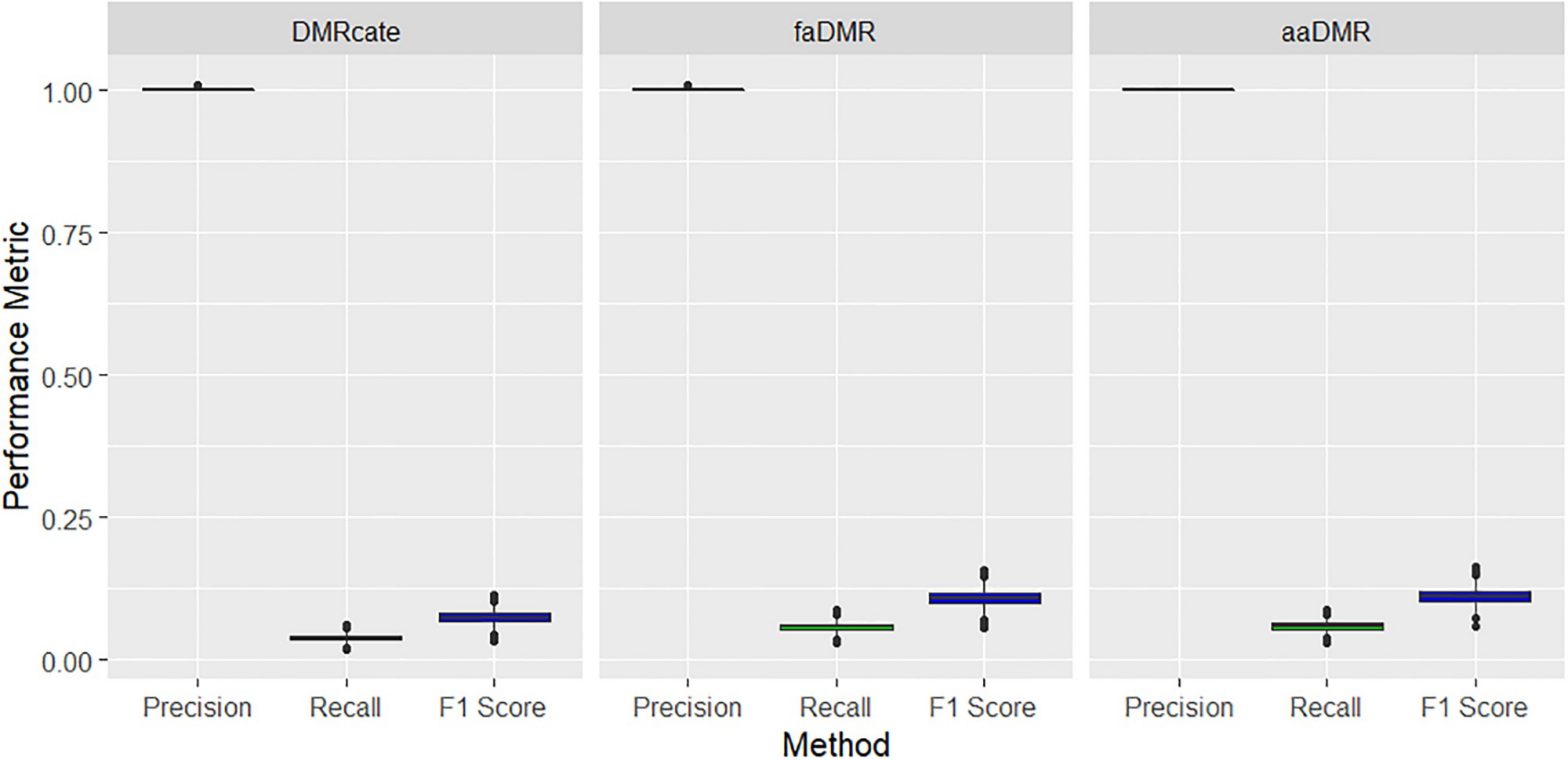
Small treatment effect (Δ*β* = 0.09): Precision, recall and F1 score metrics based on AO criteria. Boxplots of results across the 1000 simulated data sets. All pairwise comparisons of the methods are statistically significant with p < 0.001 using Tukey’s multiple comparison test from a one-way ANOVA.

**Fig 7:**
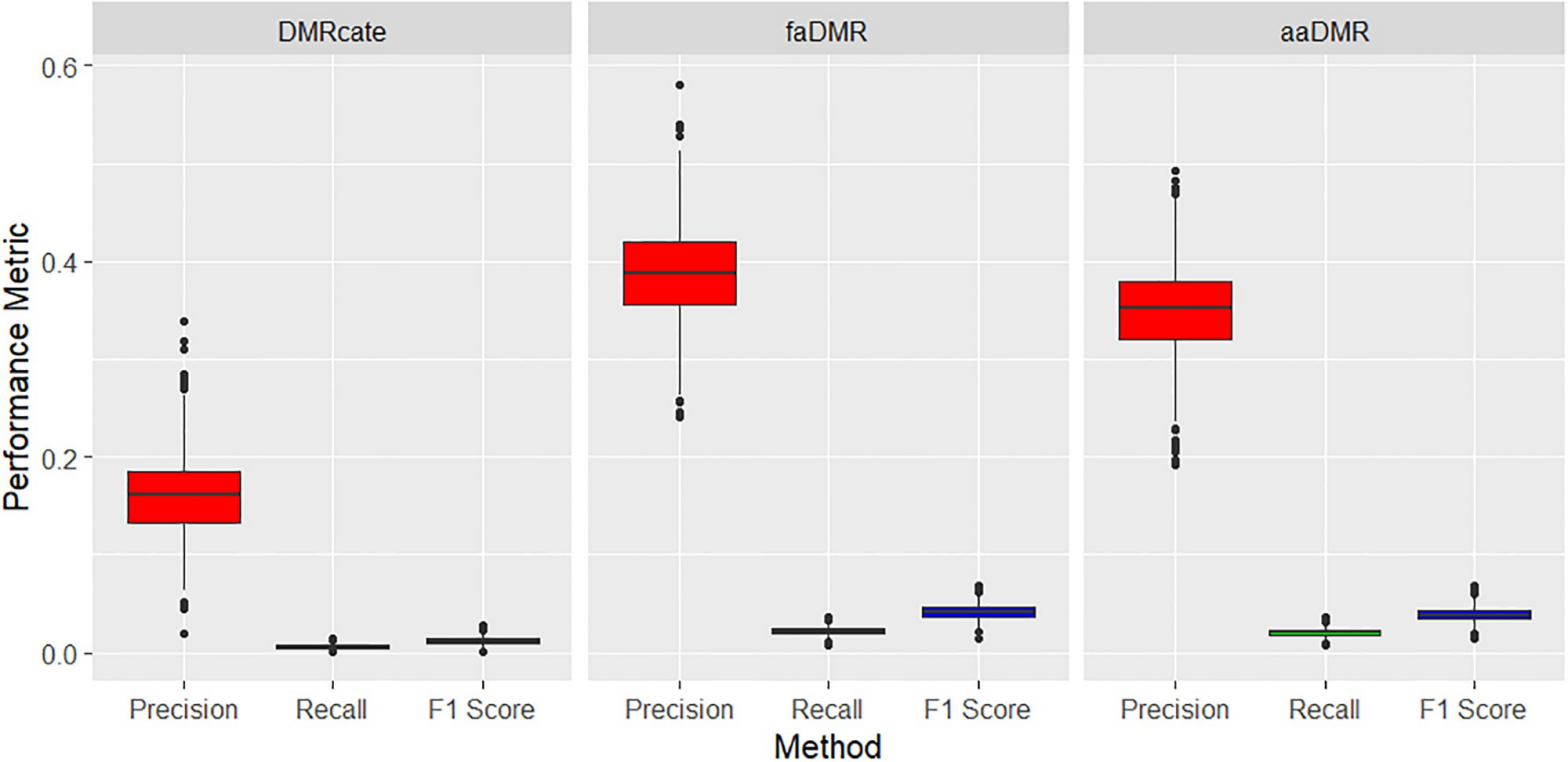
Small treatment effect (Δ*β* = 0.09): Precision, recall and F1 score metrics based on EO criteria. Boxplots of results across the 1000 simulated data sets. All pairwise comparisons of the methods are statistically significant with p < 0.001 using Tukey’s multiple comparison test from a one-way ANOVA.

For the small methylation difference (treatment effect) of Δ*β* = 0.09, all methods performed well on precision (based on the AO criteria), but very poor in terms of power. This is usually the case with power for small treatment effects. However, we highlight that our DMR detection methods performed relatively better in terms of power than *DMRcate* (see Table 3 and Fig 6). All methods under the EO criteria performed poorly, yet our methods still do better, especially on precision with median greater than 0.35 compared to the median precision of less than 0.2 for *DMRcate* across the 1000 simulated data sets (see Fig 7).

**Table 3:** Small treatment effect (Δ*β* = 0.09): A confusion matrix comparing the results from three methods (DMRcate, faDMR and aaDMR) with true DMRs based on AO criteria averaged across 1000 datasets. Sig. means statistically significant DMRs; Not Sig. indicates the number of regions that are not statistically significant.

DMRcate	True DMR	No DMR	Total
Sig	**82**	0	**82**
Not Sig.	2054	19227	21281
Total	2136	19227	21363
faDMR	True DMR	No DMR	Total
Sig.	**123**	0	**123**
Not Sig.	2013	19227	21240
Total	2136	19227	21363
aaDMR	True DMR	No DMR	Total
Sig.	**127**	0	**127**
Not Sig.	2009	19227	21236
Total	2136	19227	21363

**Fig 8:**
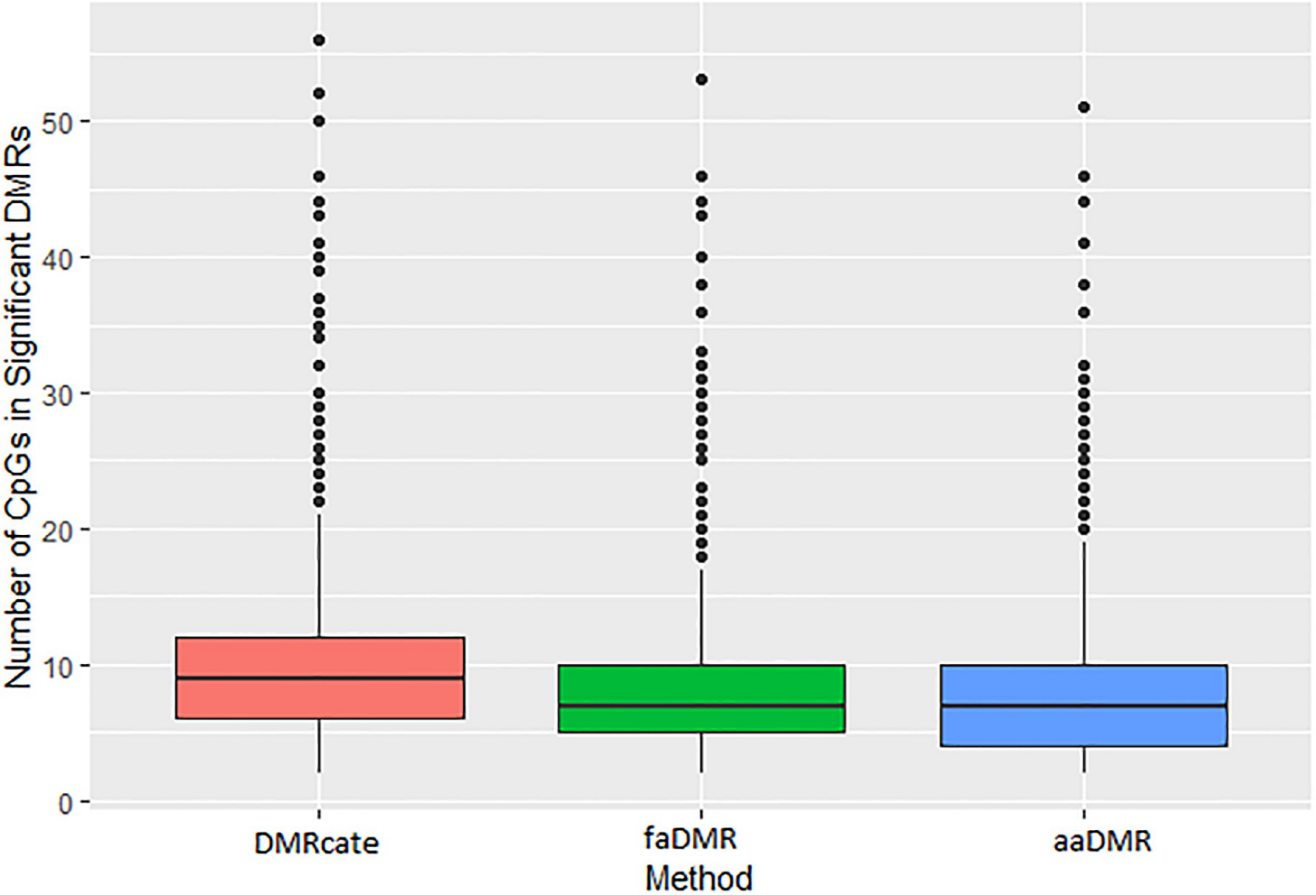
Density of CpGs in significant DMRs from one of 1000 datasets for the three methods (DMRcate, faDMR and aaDMR).

In Fig 8, we briefly compare the density of the CpGs in the DMRs obtained from one of our simulated datasets. As previously stated, these results suggest that our methods are able to detect DMRs in lower density regions compared to DMRcate.

### Real data example

#### Data extraction

We downloaded the Oral Squamous Cell Carcinoma (OSCC) data set [43] from the National Center for Biotechnology Information (NCBI) Gene Expression Omnibus (GEO) repository. The OSCC data set (with GEO accession number GSE87053) was generated using the Illumina Infinium 450K Human DNA methylation Beadchip v1.2. A total of 21 samples were collected, with 10 paired tumor and adjacent normal tissues and 1 unpaired tissue. We used the 10 paired tissues for our analysis. Information on human papillomavirus (HPV) status (5 HPV positive, 5 HPV negative) and sex (3 females and 7 males) was also provided.

#### Oral Squamous Cell Carcinoma (OSCC)

Oral cancer is the most common form of head and neck squamous cell cancer. According to the American Cancer Society, about 1 in 60 men and 1 in 140 women have a lifetime risk of developing oral cavity cancer. Aberrant DNA methylation patterns have been found to be associated with OSCC [43].

**Fig 9:**
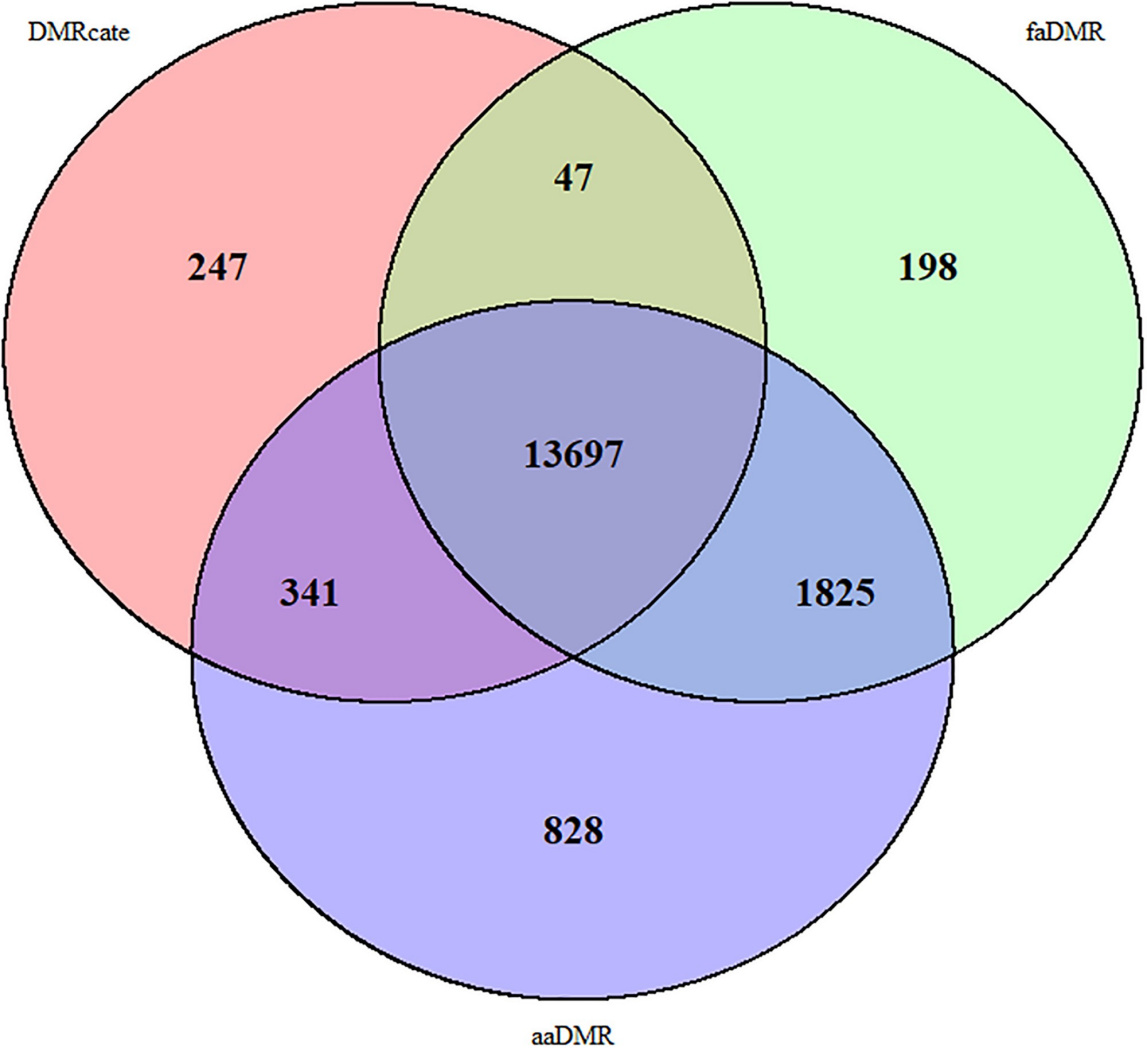
Venn diagram of significant DMRs identified by the three methods from OSCC data.

We applied our methods and *DMRcate* to the OSCC data of paired tumor and adjacent normal tissues after we had accounted for the paired data in limma’s test-statistic. We adhered to the standard quality control steps such as normalization and probe filtering using the *preprocessFunnorm* [15] function in the *minfi* R/Bioconductor package [44]. In addition to this, we controlled for the HPV status and sex in our modeling. All three methods found 13,697 DMRs. *faDMR* and *aaDMR* found more common DMRs (1825) than either method with DMRcate (341 with *aaDMR* and 47 with *faDMR*, see Fig 9). In terms of the number of uniquely identified DMRs, the results were consistent with the simulation study, as *aaDMR* detected the most DMRs (with 828 unique ones) followed by *faDMR* (with 198 unique ones) and then DMRcate (with 247 unique ones) (see Fig 9).

With the large number of DMRs detected, we sought to determine the biological relevance (i.e. the pathways enriched and the associated unique differentially methylated genes) via a KEGG pathway analysis, using the *goregion* function in the *missMethyl* package [45]. We employed this new function in the *missMethyl* package because in identifying enriched genes, it annotates probes to genes in a way that accounts for the bias towards genes with more measured CpG sites on the array (“probe-bias”) and corrects for “multi-gene bias” [45] thereby improving the type I error rate (see [45] for details). For this downstream analysis, we narrowed our discussion to comparing *aaDMR* and *DMRcate*. Whereas 30 significantly affected pathways were identified from the *aaDMR* procedure, 22 significantly affected pathways were found with *DMRcate*. Nineteen of the 22 significantly affected pathways by *DMRcate* were also reported by *aaDMR*. For the 19 pathways identified by both methods, the evidence of differentially methylated genes was stronger in *aaDMR*. We attribute this to the higher power *aaDMR* has over *DMRcate*, as shown in the simulation study. Moreover, we found that the 11 unique pathways determined by *aaDMR* were related to the immune and nervous system, suggesting that the genes affected by the OSCC disease map to these systems. In the case of *DMRcate*, the 3 unique pathways were related to immune and sensory systems, and cardiovascular disease. The associated genes from the unique pathways identified by *aaDMR* and *DMRcate* were further checked with a wide list of databases using the interactive Enrichr enrichment analysis tool [46–48]. Our analysis revealed that pathways detected by *aaDMR* contained the AKT serine-threonine protein kinase family (AKT1, AKT2 and AKT3) which has been linked to oral cancer. In their study of the specific role of AKT in OSCC, [49] revealed that the silencing of AKT1 and AKT2 genes decreased the expression of proteins regulating cancer cell survival. The frequency of appearances for the three genes (see S2 File) suggest their importance in OSCC.

### Summary of results

There were many common DMRs among the three methods compared in both the simulation study and OSCC data example. However *aaDMR* determined more unique DMRs due to its higher power. Using the EO criteria revealed that there is more room for improvement as all methods performed poorly with less than 50% precision and power for the large treatment effect and below 40% precision and 10% power for the small treatment effect. Despite this issue, we point out that our methods are more likely to detect the true length of a DMR than *DMRcate*, as our simulation results using the EO criteria reveal that *aaDMR* has better precision and recall performance. This finding supports the list of genes that are significantly affected in the pathway analysis when our methods are applied (see S2 File).

## Conclusion

The development of diseases is influenced by a number of factors, some of which are genetic in origin and some of which are environmental. Additionally, by comparing the changes in DNA methylation patterns between disease and normal samples, epigenetic markers like DNA methylation give us a way to understand how diseases are formed. However, this area is not fully understood and methods that reveal unique genomic regions and genes that are enriched by changes in methylation patterns are desirable as they provide researchers with newer areas to explore. We discovered that our method, *aaDMR*, has the capacity to provide researchers with additional pathways to investigate while also revealing particular genes that are significantly impacted by the presence of a condition, thereby assisting researchers in selecting the precise genes that need further analysis. Due to cost and resource constraints, high-throughput experiments frequently have small to medium sample sizes. This means that there is less statistical power to detect DMRs that do exist, which affects the reliability of studies and statistical data analysis results. In such cases, our method may be preferred because it has a higher statistical power to detect a DMR than *DMRcate*, especially in low treatment effect settings. Our methods use limma for the site-level statistic, which is employed in WGBS data analysis for testing for DMPs. Hence our methods can be applied to such data. In using WGBS, we recommend *aaDMR* due to the increased density of CpG sites in WGBS data. If an alternative to limma is preferred for the site-level statistic, the main additional work needed to utilize our approach would be to obtain the (approximate) statistical distribution of the locally-weighted statistic (2). Lastly, we note that our methods are validated by comparison with DMRcate, an established method previously validated using WGBS data. This comparison, leveraging DMRcate’s established validity, provides strong indirect support for the accuracy and reliability of our results.

In summary, we have developed a general class of locally-weighted estimators for use in DMR detection and shown its consistency and asymptotic normality. We have proposed the normalized kernel-weight methods within this general class, which have a higher power to detect a true DMR than *DMRcate* without sacrificing precision for large treatment effect and a higher precision than DMRcate for a low treatment effect. Essentially, our methods demonstrate two things: (1) that using the normalized kernel-weight is a better way to borrow information from neighboring sites and account for co-methylation than *DMRcate* (revealed by comparing *faDMR* with *DMRcate*) and (2) that accounting for co-methylation using the adaptive-array technique increases the susceptibility of detecting true methylation differences (revealed by comparing *aaDMR* with *faDMR*).

## Supporting information

S1 File This file contains the statement and proofs to theorems 1 and 2. It also contains details of the simulation setup.
(PDF)

S2 File This file contains list of genes produced from the pathway analysis based on DMRcate, faDMR and aaDMR. (CSV)

## Abbreviations

450K: Infinium HumanMethylation450 BeadChip
aaDMR: Array-adaptive DMR
AO: Any Overlap
BH: Benjamini-Hochberg
CHOL: Cholangiocarcinoma
DMR: Differentially methylated regions
EO: Equal Overlap
EPIC: Infinium HumanMethylationEPIC BeadChip
EWAS: Epigenome-wide association studies
faDMR: Fixed-spacing array DMR
FDR: False Discovery Rate
FN: False negatives
FP: False positive
GEO: Gene Expression Omnibus
KEGG: Kyoto Encyclopedia of Genes and Genomes
NCBI: National Center for Biotechnology Information
OSCC: Oral Squamous Cell Carcinoma
TCGA: The Cancer Genomic Atlas
TP: True positive
WGBS: Whole-Genome Bisulfite Sequencing
