# Supplementary material for: Differential methylation region detection via an array-adaptive normalized kernel-weighted model
Source: PLoS One. 2024 Jun 28;19(6):e0306036. doi: 10.1371/journal.pone.0306036 (PMC11213316; doi:10.1371/journal.pone.0306036)
Supplement: S1 File — It also contains details of the simulation setup. (PDF) [file pone.0306036.s001.pdf]

Differential Methylation Region Detection via an Array-adaptive Normalized Kernel-weighted Model Supplementary Text

Daniel Alhassan Gayla R. Olbricht Akim Adekpedjou

1 Asymptotic Behavior of Our Proposed Statistic

This subsection, pertains to the long-term behavior of a novel statistic, $S(x_i)$, which is pivotal in site-level testing. The focus here is on two fundamental properties of this statistic: its consistency and its convergence to normality under certain conditions.

Theorem 1 (Consistency)**.** *This aspect pertains to the reliability of $S(x_i)$ over repeated applications, a critical feature in scientific studies. The statistic is formulated as a weighted sum of independent F-distributed random variables, commonly employed in genomics research. The weights assigned to these variables (w_j) are chosen to reflect their relative importance in the sum. Theorem 1 assures that under specific mathematical conditions, our statistic remains consistent, meaning it provides stable results across different instances.*

Let $\{Y_j,\ j > 1\}$ be independent F-distributed random variables obtained from site-level testing via limma's moderated F-statistic (t^2-statistic) and $\{w_j,\ j > 1\}$ be constants satisfying $\sum_{j=1}^{n} w_j^2 = \mathcal{O}\, n w_n^2$. Further, at CpG site x_i, let $S(x_i) = \sum_{j=1}^{n} w_j(x_i) Y_j$ where

$$w_j(x_i) = \begin{cases} 1, & j = i \\ t \in (0,1), & j \neq i \end{cases}.$$

Then,

$$\frac{\sum_{j=1}^{n} w_j \left(Y_j - EY\mathbf{1}_{\{|Y| \leq n^2\}}\right)}{w_n n^2} \xrightarrow{p} 0.$$

Theorem 2. *This addresses a central concept in statistics: as the number of data points increases, the distribution of our statistic approaches a normal distribution. The theorem is based on the same weighted sum of F-distributed random variables as in Theorem 1. However, here the focus is on how this sum behaves as the sample size (the neighboring sites) increases, demonstrating a convergence to a normal distribution.*

Let $\{Y_j,\ j > 1\}$ be independent F-distributed random variables obtained from site-level testing via limma's moderated F-statistic (t^2-statistic) and let $\{w_j,\ j > 1\}$ be constants satisfying $0 < w_j < 1$ with $\sum_{j=1}^{n} w_j = 1$. At CpG site x_i for observed y_i, define $S(x_i) = y_i + \sum_{j \neq i}^{n} w_j Y_j$. Then

$$y_i + \frac{\sum_{j \neq i}^{n} w_j \left(Y_j - E(Y)\right)}{\sqrt{\sum_{j \neq i}^{n} w_j^2 \sigma_j^2}} \xrightarrow{d} y_i + Z \quad \text{as} \quad n \to \infty \tag{1}$$

where $Var\left(\sum_{j \neq i}^{n} w_j Y_j\right) = \sum_{j \neq i}^{n} w_j^2 \sigma_j^2$ and $Z \sim N(0,1)$.

Theorems 1 and 2 apply to a specific case of the general locally-weighted statistic.

2 Proof of Asymptotic Results

This section pertains to the proof of the asymptotic results of our proposed estimator $S(x_i)$. More specifically we investigate the situation under which $S(x_i)$ is consistent and obeys the central limit theorem (CLT).

2.1 Proof of Theorem 1

Proof. To reduce notational complexity, we write $S(x_i)$ as S and $w_j(x_i)$ as w_j. We will take Y to be χ^2 distributed for the basis of our context. For $n > 1$, $S(x_i)$ has $(n-1)$ weighted Y_i's and one unweighted Y_i. Since Y_i's are iid we can write (2) , without loss of generality:

$$
\begin{aligned}
S &= Y_1 + w_2 Y_2 + \cdots + w_{n-1}Y_{n-1} + w_n Y_n \\
&= Y_1 + \sum_{j=2}^{n} w_j Y_j \\
&= \sum_{j=1}^{n} w_j Y_j \qquad (2)
\end{aligned}
$$

where $\sum_{j=2}^{n} w_j = 1$ and $w_1 = 1$.

Let $Y \sim \chi_1^2$ (see section 3 for proof). Define $b_n = w_n n^2$ so that $c_n = n^2$ where $0 < w_j \leq 1$.

$$
\begin{aligned}
\lim_{n\to\infty} nP(Y > n^2) &\leq \frac{nE(Y)}{n^2} \qquad \text{(By Markov Inequality)} \\
&= \frac{E(Y)}{n} \\
&= \frac{1}{n} \to 0 \text{ as } n \to \infty
\end{aligned}
$$

$\implies nP(Y > n^2) = \text{o}(1)$. Now,

$$
\begin{aligned}
\frac{n^2}{n} &= n \uparrow \text{ for } n > 1 \\
\frac{\sum_{j=1}^{n} w_j^2}{n w_n^2} &\leq \frac{n}{n w_2^2} \\
&= \frac{1}{w_n^2}
\end{aligned}
$$

$\implies \sum_{j=1}^{n} w_j^2 = \mathcal{O}\left(\frac{1}{w_n^2}\right)$.

By the Lemma of Adler and Rosalsky (1991) we have that,

$$
\sum_{j=1}^{n} w_j^2 E\left(Y^2 \mathbf{1}_{\{|Y| \leq n^2\}}\right) = \text{o}(w_n n^2) \qquad (3)
$$

and by Theorem 1 of Adler and Rosalsky (1991), we have that

$$
\frac{\sum_{j=1}^{n} w_j \left(Y_j - EY\mathbf{1}_{\{|Y|\leq n^2\}}\right)}{w_n n^2} \xrightarrow{p} 0. \qquad (4)
$$

□

Definition 1 (Uniform Tightness)**.** *A collection of random variables* $\{X_\alpha\}_{\alpha \in A}$ *is uniformly tight if* $\forall \epsilon > 0$, *there exists* $M < \infty$ *such that:*

$$
\sup_{\alpha} P(|X_\alpha| \geq M) \leq \epsilon.
$$

Lemma 1. $\{Y_{1,v}; v \geq 1\}$ *be a family of F-distributed random variables with degrees of freedom 1 and* v, *i.e.* $Y \sim F_{1,v}$. *Then the family is uniformly tight.*

Proof. $E(Y) = \frac{v}{v-2}$ for $v \geq 3$. $\exists\, v_0$ such that $\frac{v}{v-2} \leq 2$. $\forall \epsilon > 0$, $\exists\, M_1$ such that $P(Y_{1,1} \leq M_1) > 1 - \frac{\epsilon}{2}$ and $P(Y_{1,2} \leq M_1) > 1 - \frac{\epsilon}{2}$. Hence $Y_{1,1}$ and $Y_{1,2}$ are tight. Now, $\forall v \geq v_0$, $P(Y_{1,v} \geq M) \leq \frac{E(Y_{1,v})}{M} \leq \frac{3}{M}$. For $\frac{3}{M} < \epsilon$, we have that $\forall v \geq v_0$, $P(Y_{1,n} > M_\epsilon) < \epsilon$. Set $M_0 = \max(M_1, M_\epsilon)$ we have $\sup_{v \geq v_0} P(Y_{1,v} < M_0) > 1 - \epsilon$. □

2.2 Proof of Theorem 2

Proof. It suffices to show that

$$\frac{\sum_{j\neq i}^{n} w_j \left(Y_j - E(Y)\right)}{\sqrt{\sum_{j\neq i}^{n} w_j^2 \sigma_j^2}} \xrightarrow{d} Z. \tag{5}$$

Define $U_j = w_j \left(Y_j - E(Y)\right)$ so that $E(U_j) = 0$ and $Var(U_j) = \sum_{j\neq i}^{n} w_j^2 \sigma_j^2 := s_n^2$.

Applying Lyapunov's CLT condition (Billingsley, 1986; Resnick, 1999) and taking $\delta = 1$ we only need to show that

$$\sum_{j=1}^{n} \frac{E|U_j|^3}{s_n^3} \to 0. \tag{6}$$

To complete the proof we need the concept of uniform tightness. See Definition 1, Lemma 1 and the proof that shows the family of F distributed random variables is uniformly tight.

By Lemma 1, U_j is uniformly bounded by M_0. Then,

$$\sum_{j=1}^{n} \frac{E|U_j|^3}{s_n^3} \leq \sum_{j=1}^{n} \frac{M_0 E|U_j|^2}{s_n^3} = \frac{M_0}{s_n} \to 0 \text{ as } n \to \infty.$$

□

3 Miscellaneous Result

Corollary 1. *Let $Y \sim F_{(1,\nu)}$. Then as $\nu \to \infty$,*

$$Y \xrightarrow{d} \chi_1^2 \tag{7}$$

where χ_1^2 is a chi-squared distribution with 1 degree of freedom.

Proof. Define $F = \dfrac{U/\mu}{V/\nu}$. If U be a chi-square random variable with μ degrees of freedom, V be a chi-square random variable with ν degrees of freedom and U and V be independent then by definition F is an F-random variable. It only suffices to show that with $\mu = 1$, as $\nu \to \infty$, $\frac{V}{\nu} \xrightarrow{p} 1$. By Chebychev's inequality,

$$\begin{aligned} P\left(\left|\frac{V}{\nu} - 1\right| > \varepsilon\right) &\leq \frac{E\left(\frac{V}{\nu} - 1\right)^2}{\varepsilon^2} \\ &= \frac{E(V-\nu)^2}{\nu^2\varepsilon^2} \\ &= \frac{Var(V)}{\nu^2\varepsilon^2} \\ &= \frac{2}{\nu\varepsilon^2} \to 0 \qquad as \qquad \nu \to \infty. \end{aligned}$$

The result, $Y \xrightarrow{d} \chi_1^2$ follows by Slutsky's theorem. □

4 Steps to Obtain CHOL data from The Cancer Genome Atlas Program (TCGA)

We outline briefly steps to obtaining the Cholangiocarcinoma (CHOL) data set used in our simulation.

- Use the link (https://portal.gdc.cancer.gov/repository) to access the TCGA GDC data portal repository.

- Under the `Cases` tab on the left, select `TCGA` under Program. Select `TCGA-CHOL` from Project section.
- Under the `Files` tab and section Data Format, select `idat`. You should see 90 idat files.
- Download the manifest file and using the GDC transfer tool, download data.

After cleaning, only 18 sample names matched which resulted in 36 idat files.

5 Description of Simulated Data

We simulated our data in a manner similar to Peters et al. (2015); therefore, we refer the reader to the supplementary text of Peters et al. (2015), as the simulation description outlined in this paper is based on their ideas. We outline these below:

1. On the basis of the 450K array containing 485,512 probes, we constructed an empirical methylation data set.

   (a) Methylated and Unmethylated Probes
   Probes were classified as fully methylated or unmethylated using TCGA data (n = 18 samples) on bile duct cancer (CHOL) (see section 4). Probes with an average beta greater than 0.5 after normalization using the functional normalization method (Fortin et al., 2014) were classified as fully methylated; otherwise, they were classified as unmethylated. The fully methylated to unmethylated ratio was 55.5% to 44.5%.

   (b) Define Candidate Differentially Methylated Regions (DMRs)
   The candidate DMRs were defined as genomics regions with probes annotated as "TSS200" or "TSS1500" that were no more than 1000 base pairs apart. We obtained 21,363 candidate DMRs with probe counts ranging from 1 to 88, with a median of 6 probes and an average of 6.55 probes.

2. For each simulated data set, we randomly assigned 5% of the 21,363 (i.e., 1068) candidate DMRs to be true hypermethylated DMRs and 5% to be true hypomethylated DMRs. The remaining 90% of the candidate regions were not true DMRs. We set the true methylation difference to be 0.2 (a large methylation difference) as do Peters et al. (2015) and also investigate a small methylation difference equal to 0.09. We simulated random values from a uniform distribution to represent the mode of a beta distribution as follows. We generated two values from the uniform distribution to act as beta modes for each region, one for treatment and one for control: beta1 $\sim$ Uniform(0.01, 0.79) and beta2 = beta1 + 0.2. As a result, beta2 $\sim$ Uniform (0.21, 0.99). For hypermethylated regions, the control samples' base methylation level was set to beta1, and the treatment samples' base methylation level was set to beta2. For hypomethylated regions, this allocation was reversed.

3. For the probes inside the selected DMRs, we simulate $\beta-$values representing the proportion of methylation for control and treatment samples from beta (a, b) with a and b obtained using the mode in step (2) and $a + b + 2 = K = 100$. This value was selected as a level of variability in the sampling distribution that is consistent with reality (Peters et al., 2015). Given K and the mode, the following R code simulates the random $\beta-$values:

   ```
   r <- mode/(1 - mode)
   B <- K/(1+r)
   A <- r*B
   a <- A + 1
   b <- B + 1
   beta <- rbeta(a=a, b=b)
   ```

   Source: Adapted from Peters et al. (2015)

4. For the probes outside the selected DMRs, we generate $\beta-$values from two beta distributions using the methylated or unmethylated status described in step 1. We sample from `rbeta(a = 14, b = 3.12)` and `rbeta(a = 2, b = 11.11)`. These values of a and b were chosen to be reasonably close to Peters et al. (2015).

5. The aforementioned steps yielded a data set of 485,512 rows and 20 columns (10 treatment, 10 controls). The entire simulation was repeated 1000 times.

References

Adler, A. and Rosalsky, A. (1991). On the weak law of large numbers for normed weighted sums of iid random variables. *International Journal of Mathematics and Mathematical Sciences*, 14(1):191–202.

Billingsley, P. (1986). *Probability and Measure*. John Wiley and Sons, second edition.

Fortin, J.-P., Labbe, A., Lemire, M., Zanke, B. W., Hudson, T. J., Fertig, E. J., Greenwood, C. M., and Hansen, K. D. (2014). Functional normalization of 450k methylation array data improves replication in large cancer studies. *Genome Biology*, 15(12):503.

Peters, T. J., Buckley, M. J., Statham, A. L., Pidsley, R., Samaras, K., V Lord, R., Clark, S. J., and Molloy, P. L. (2015). De novo identification of differentially methylated regions in the human genome. *Epigenetics Chromatin*, 8:6.

Resnick, S. I. (1999). *A probability path*. Birkhäuser Boston.
